# The association between neighbourhood walkability and blood lipids: a Canadian population study

**DOI:** 10.1186/s12944-024-02267-x

**Published:** 2024-09-12

**Authors:** Sabit Cakmak, Anna Lukina, Robert Dales

**Affiliations:** 1 Environmental Health Science and Research Bureau, Health Canada, 251 Sir Frederic Banting Driveway, Ottawa, ON K1A 0K9 Canada; 2 University of Ottawa and Ottawa Hospital Research Institute, Ottawa, Canada

**Keywords:** Walkability, Lipid metabolism, Fine particulate matter, Canadian health measures survey, Public health

## Abstract

We examined the association between walkability and blood lipids in a nationally representative sample of 29,649 participants aged 3–79 years who participated in the Canadian Health Measures Survey (CHMS) cycles 1 to 6. We focused on seven lipid biomarkers: apolipoprotein A (Apo A), apolipoprotein B (Apo B), triglycerides (TG), high-density lipoprotein cholesterol (HDL), low-density lipoprotein cholesterol (LDL), total cholesterol (TC), and TC/HDL. Cross-sectional associations were analyzed using generalized linear mixed models incorporating survey-specific sampling weights. An increase in the Canadian Active Living Environments Index, a measure of neighborhood walkability, equivalent to the magnitude of its interquartile range (IQR) was associated with the following percentage (95% confidence intervals (CI)) changes in lipids: decreased TG, -2.85 (-4.77, -0.93) and TC/HDL, -1.68 (-2.80, -0.56), and increased HDL, 1.68 (0.93, 2.42). Significant effects were largely restricted to adults (aged 17 to 79). In the younger age group there were no significant associations between walkability and lipids in the fully adjusted model. Significant associations were more frequently seen in females than males. For females, fully adjusted significant inverse associations were observed for TG, LDL, and TC/HDL, and there were positive associations with HDL and Apo A. Canadians living in more walkable neighborhoods have more favorable lipid profiles, suggesting that the built environment has the potential to influence the risk profile for cardiovascular health, especially among adults and females.

**Supplementary Information:**

The online version contains supplementary material available at 10.1186/s12944-024-02267-x.

## Highlights

Blood lipids were measured in participants of the Canadian Health Measures Survey.

Neighborhood walkability was measured by the Canadian Active Living Index.

Increased walkability was associated with lower TG and TC/HDL and higher HDL.

Favourable associations were more common in females.

Increased walkability may improve cardiovascular risk profiles.

**Supplementary Information:**

The online version contains supplementary material available at 10.1186/s12944-024-02267-x.

## Introduction

Cardiovascular disease (CVD) is the major cause of mortality in many developed countries resulting in nearly 18 million deaths annually [1]. In Canada, CVD mortality is the second leading cause of mortality after cancer, and men are twice as likely to suffer from heart disease than women [2]. Blood lipid concentrations are positively associated with the risk of developing CVD and all-cause mortality, as shown in a systematic review covering 11 studies from South Korea, China, and America [3]. Apart from personal risk factors, such as tobacco smoking, alcohol consumption, and unhealthy diet or genetics, characteristics of the built neighbourhood environment may possibly influence cardiovascular health through encouraging physical activities, such as walking and cycling [4, 5]. A walkable neighbourhood is one which facilitates access to public transport, parks, playgrounds, businesses, retail shopping, and other points of interest. This may encourage more physical activity which could benefit lipid levels and overall cardiovascular health, but there are concerns that increased walkability could facilitate access to unhealthy food outlets [6, 7], and the increased population density often associated with walkability may lead to increased exposure to traffic-related ambient air pollution [8, 9] and possibly increased environmental noise [10, 11].

Previous studies investigating the associations between neighbourhood walkability and blood lipid profiles have yielded variable results with some finding inverse associations between elevated blood lipids and walkability [12–14] and others not [15–18]. Chandrabose et al. [13] reported an inverse association between walkability and high-density lipoprotein (HDL) but not triglycerides (TG), while de Courreges et al. [16] found no association between walkability and TG, HDL, and low-density lipoprotein (LDL). One cohort longitudinal analysis found no effect on a measure of dyslipidemia comprised of increased TG, decreased HDL and being prescribed lipid lowering agents [19] while another longitudinal study found a significant positive association with TG [15]. A cross-sectional analysis of data collected from 78,023 participants living in Toronto, Canada found positive correlations between the Walk Score^®^ and total cholesterol (TC) and HDL, but not TG or LDL [12]. An analysis of the CHMS cycle 1 data revealed a negative association with walkability and TC/HDL but the interquartile linear trend was not significant [20]. To our knowledge, the effect of walkability on apolipoprotein B (Apo B), a major indicator of lipid-related risk for CVD [21, 22, 23], and on lipid profiles in children [24, 25], has not been previously reported.

The present cross-sectional Canadian population-based study investigates the association between neighbourhood walkability measured by the Canadian Active Living Index (Can-ALE) and lipid profiles including Apo B, in both children (3 to ≤ 16 years old) and adults (17 to 79 years old).

## Methods and materials

### Design and sample

The nationally representative cross-sectional Canadian Health Measures Survey (CHMS) has been carried out in two-year cycles since 2007, with a total of six cycles completed between 2007 and 2019. Each cycle resampled the population. The CHMS population has been previously described in detail [26–30]. Blood sampling for lipids was done at one of 15 collection sites stratified by five regions sampled from approximately a possible 425 sites. The target population for our study is 6 to 79 year old residents of the ten Canadian provinces. Simple random sampling stratified by age group was done for each site. Survey weights were assigned to each participant proportional to the number of people in the population represented by that person [31]. The present study included CHMS participants from cycle 1 (2007–2009) through cycle 6 (2018–2019), who had at least one lipid biomarker and a neighbourhood active living index calculated. Participants who were taking lipid-lowering or diabetes medications were excluded from analyses.

Ethics approval for all components of the CHMS was obtained by the Health Canada and Public Health Agency of Canada Research Ethics Board (REB#2005-0025). Participation in the CHMS is voluntary. Informed consent was obtained for those 14–79 years old, a parent or legal guardian consented for children between 3 and 13 years, and children aged 6–13 provided assent. The air quality data used in this study were publicly available and no ethics approval was required for the data linkage.

### The Canadian active living environments database (Can-ALE)

Variables in the Canadian Active Living Environments (Can-ALE) database [32] represent characteristics of Canadian neighbourhoods that encourage active living activities, such as walking, cycling, and taking public transit which will be referred to “walkability” or “active living” in the present study. From the 2016 Can-ALE database, two composite variables representing walkability were used in the present study. ALE_INDEX (ALE) is the continuous sum of the z-scores of intersection density, dwelling density, and points of interest and ALE_TRANSIT (ALE + T) is the sum of the z-scores of ALE_INDEX plus transit stops or stations [33]. We have previously described the use of ALE + T [34]. Larger values indicate increased walkability. The measures address a one-kilometer perimeter around the center of a dissemination area (DA), a geographic unit representing between 400 and 700 people. Transit measures were available for 35,338 DAs in the larger cities, whereas other measures of walkability were available for approximately 56,000 DAs across Canada. The 5th and 95th percentile z-score ranges were − 2.12 and 4.83 for ALE, and − 3.12 and 6.05 for ALE + T. The ALE_INDEX was documented to be strongly correlated (*r* = 0.78) with questionnaire-ascertained reports of getting to work by walking, cycling, or using public transportation [32]. DA were linked to participants using their 6-digit postal codes. In addition to the 2016 Can-ALE data used in the present study, there exists a 2006 Can-ALE database which contained only intersection and dwelling density.

To test the hypothesis that ALE and ALE + T were stable over time, we calculated the Pearson correlation coefficients for z-scores of each of ALE and ALE + T between 2006 and 2016 across all DAs in our study sample. The results indicated high correlations for both measures: *r* = 0.84 for ALE (*p* < 0.001) and *r* = 0.85 for ALE + T (*p* < 0.001).

### Blood lipids

The present study focused on seven lipid biomarkers: TC, LDL, HDL, TC/HDL, TG, Apo A, and Apo B measured in blood serum. Individuals between 3 and 79 years old were eligible for blood collection. TG, LDL, Apo A, and Apo B were always collected after fasting, while HDL and TC were collected in both the fasting and non-fasting states. The age range for sampling TC and HDL was 3 to 79 years, and the range was 6 to 79 years for Apo A, Apo B, TG, and LDL. Lower levels of TC, LDL, TC/HDL, TG, and Apo B, and higher concentrations of HDL and Apo A are favorably associated with a reduced risk of CVD [35].

### Covariates

These included age, sex, race/ethnicity, highest household education (less than secondary school graduation, some secondary but no post-secondary degree/diploma, some post-secondary education, post-secondary degree/diploma), total household income (quartiles), active smoking (current, former, never), environmental tobacco smoke exposure (ETS) (exposure to smoking in the home at least once during the past month, other), alcohol consumption (regular, occasional, former, never), province of residence, annual average temperature, and mean 24-hour ambient exposure to fine particulate air pollution < 2.5 micrometers in median aerodynamic diameter (PM_2.5_). Covariates were self-reported with the exceptions of temperature and PM_2.5_. Information on active smoking and alcohol were only collected for those ≥ 12 years old. Missing variables were assigned to a category which was included in the analysis to avoid loss of subjects which could otherwise occur with only one missing covariate.

### Statistical analysis

We used three models to examine the associations between walkability measurements (ALE and ALE + T) and individual lipid biomarkers. Model 1 tested the association between walkability and individual lipids using linear regression models with sampling weights applied to all models. Province of residence, the walkability index z-scores and selected lipid were the only variables in the model.

Model 2 was a generalized linear mixed model adjusted for self-declared sex, age, race/culture, income, education (Table 1) and survey cycle, while treating province of residence as a random effect, as previously described [36, 37].

**Table 1:** Characteristics of the CHMS study participants (cycles 1 to 6 combined) overall and by age group (*≤* 16 years, > 16 years) and self-declared sex (male, female)

Mean ± SD or *n* (%)					
Variable	All Ages	Age *≤* 16 years	Age > 16 years	Male	Female
**Number of participants included**	26,063	9675	16,388	12,807	13,256
**Age (years)**	34.22 ± 22.44	9.37 ± 3.86	45.70 ± 17.38	31.93 ± 22.45	32.49 ± 22.42
**Self-declared sex**:					
Male	12,807 (49.14)	4902 (50.67)	7905 (48.24)	12,807 (100)	na
Female	13,256 (50.86)	4773 (49.33)	8483 (51.36)	na	13,256 (100)
**Race/ethnicity**:					
White	19,982 (76.67)	6853 (70.83)	13,129 (80.11)	9851 (76.92)	10,131 (76.43)
Non-white	6081 (23.33)	2822 (29.16)	3259 (19.88)	2956 (23.09)	3125 (23.57)
**BMI (kg/m** ^**2**^ **)**	24.15 ± 6.76	18.70 ± 4.23	27.39 ± 5.84	24.22 ± 6.45	24.07 ± 7.05
**Total Annual Household Income**:					
First quartile (<$42,000)	6045 (23.19)	1770 (18.29)	4275 (26.09)	2690 (21.00)	3355 (25.31)
Second quartile ($42,000-<$74,200)	6362 (24.41)	2250 (23.26)	4112 (25.09)	3177 (24.81)	3185 (24.03)
Third quartile ($74,200-$115,000)	6494 (24.92)	2687 (27.77)	3807 (23.23)	3319 (25.92)	3175 (23.95)
Fourth quartile (>$115,000)	5781 (22.18)	2546 (26.32)	3235 (19.74)	3013 (23.53)	2768 (20.88)
Missing	1381 (5.30)	422 (4.36)	959 (5.85)	608 (4.75)	773 (5.82)
**Highest Household Educational attainment**:					
< Secondary school graduation	1478 (5.67)	322 (3.33)	1156 (7.05)	637 (4.97)	841 (6.34)
Secondary school education	2949 (11.31)	978 (10.11)	1971 (12.03)	1436 (11.21)	1513 (11.41)
Some post-secondary education	725 (2.78)	231 (2.39)	494 (3.01)	355 (2.77)	370 (2.79)
Post-secondary degree or diploma	20,075 (77.02)	7795 (80.57)	12,280 (74.93)	9938 (77.60)	10,137 (76.47)
Missing	836 (3.21)	349 (3.61)	487 (2.97)	441 (3.44)	395 (2.98)
**Cigarette smoking status**:					
Current	2595 (9.96)	511 (5.28)	2084 (12.72)	1305 (10.19)	1290 (9.73)
Former	738 (2.83)	172 (1.78)	566 (3.45)	360 (2.81)	378 (2.85)
Never	16,270 (62.43)	4717 (48.75)	11,553 (70.50)	7945 (62.04)	8325 (62.80)
Other	6460 (24.78)	4269 (44.18)	2185 (13.33)	3193 (24.96)	3263 (24.62)
**Alcohol consumption**:					
Regular	11,462 (43.98)	2552 (26.38)	8910 (54.37)	5880 (45.91)	5582 (42.11)
Occasional	3205 (12.30)	884 (9.14)	2321 (14.16)	1382 (10.79)	1823 (13.75)
Former	1908 (7.32)	466 (4.82)	1442 (8.80)	914 (7.14)	994 (7.50)
Never	3018 (11.58)	1494 (15.44)	1524 (9.30)	1430 (11.17)	1588 (11.98)
Other	6470 (24.83)	4279 (44.22)	2191 (13.36)	3201 (24.99)	3269 (24.66)
**Home ETS**					
No	23,482 (90.10)	8885 (91.83)	14,597 (89.07)	11,493 (89.74)	11,989 (90.44)
Yes	2475 (9.50)	774 (8.00)	1701 (10.38)	1253 (9.78)	1222 (9.22)
Missing	106 (0.41)	16 (0.17)	90 (0.55)	61 (0.48)	45 (0.34)

na-not applicable

Home ETS-Exposure to Environmental Tobacco Smoke at home at least once during the past month

Model 3 included variables used in Model 2 and additionally included current cigarette smoking, environmental tobacco smoke, alcohol consumption, and mean PM2.5, measured by the National Air Pollution Surveillance System. Information on active smoking and alcohol was only collected for those ≥ 12 years old.

Findings were presented for the overall study group and by age (≤ 16, > 16 years old) and sex (female, male). In adjusted analyses stratified by age and sex, age was adjusted for when stratified by sex, and sex was adjusted for when stratified by age. Results were expressed as the percentage change in serum lipid biochemical markers for an increase equivalent to the magnitude of the interquartile range (IQR) in the walkability measure. In addition to dichotomizing age, we assessed the effect of age in more detail by smoothed plots using penalized β-splines. All data management and regression modeling were performed either using SAS Enterprise Guide 7.1 [38] or R version 4.0.2 [39].

## Results

### Study participants

Descriptive statistics of the CHMS study participants are presented in Table 1. Of 35,164 participants in the six data collection cycles, 29,649 participants had at least one blood lipid marker and at least one neighborhood walkability measurement. The present study sample size was 26,063 after excluding those (*n* = 3,586) taking medications for diabetes and dyslipidemia. Missing observations for any characteristics described in Table 1 were assigned to a category which was included in the analysis to avoid loss of subjects which could otherwise occur with only one missing covariate. We compared the group analyzed (*n* = 26,063) with those excluded because of taking lipid or diabetes medications, or missing a walkability measure or lipid value (*n* = 9,101). The differences in prevalences of characteristics listed in Table 1 were within 10% for all variables. The largest differences (study group versus excluded) were: White (76.7% in study group versus 68.9% in excluded), a post-secondary degree or diploma (77.0% versus 84.8%), never smokers ( 62.4% versus 55.2%), regular alcohol (44.0 versus 39.2), and no home ETS exposure (90.1 versus 96.1).The number of observations available for multivariate analysis varied by lipid biomarker because some were collected only in a subsample. Sample sizes by biomarker were 26,063 for HDL, 26,061 for TC and TC/HDL, 11,777 for TG, 11,713 for LDL, 6,313 for Apo A, and 6,303 for Apo B.

Most participants were Caucasian (79.0%), non-smokers (62.6%) and had a post-secondary degree or diploma (77.7%). 43.8% consumed alcohol regularly. Compared to adults, children had a lower BMI and lived in households with higher incomes and educational attainment. Males and females had similar sociodemographic profiles. The neighborhood estimated annual average of 24-hour mean PM_2.5_ concentration which was 6.6 µg/m^3^ (Standard Deviation (SD): 2.2) for all participants, 29,649, based on a 6-digit postal code (Table 2).

**Table 2:**
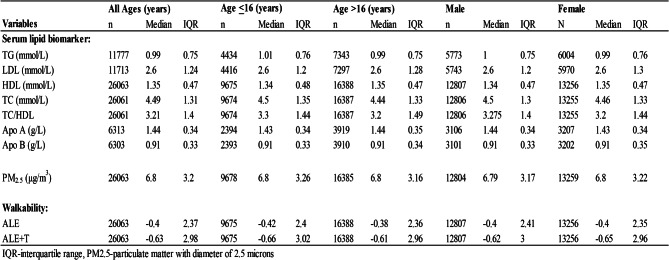
Median and IQR of serum lipid biochemical measures, walkability indexes and ambient PM_2.5_ for CHMS study participants separated by age and sex

Total household income and highest household education were stratified by categorical levels of walkability defined in the Can-ALE database for ALE and ALE + T. The variables used for stratification were ALE_CLASS and ALE_TRANSIT_CLASS with scores 1(low) to 5 (high) (https://canue.ca/wp-content/uploads/2018/03/CanALE_UserGuide.pdf). Maximum and minimum percentages by characteristic between classes/strata were: 36–38% for those ≤ 16 years old, 49-50% for males, 76-77% for the highest household education group, and 21-23% for the highest household income group (Table S1). The data indicate a consistent distribution of sociodemographic factors across different walkability levels. Categories of city size defined by Metropolitan census or agglomeration areas were stratified by ALE_CLASS. An ALE score of 1 varied between 33% and 36% by population size, and an ALE score of 5 varied between 5% and 6%, indicating that city size was not strongly related to the distribution of ALE classes (Table S2).

### Lipid metabolism markers

The median value and IQR of each serum lipid biochemical measure for CHMS study participants used in the analyses are shown in Table 2. The number of each test available varied because of the different age groups eligible for the test. The median concentration of TG detected was 0.76mmol/L in children and 1.13mmol/L in adults. Median HDL was slightly lower in males (1.25mmol/L) than females (1.42mmol/L) and median Apo A was slightly higher in females (1.52 g/L) than males (1.36 g/L). Median TC/HDL was lower in children (2.9) compared to older participants (3.5).

### Walkability index

**Fig. 1:**
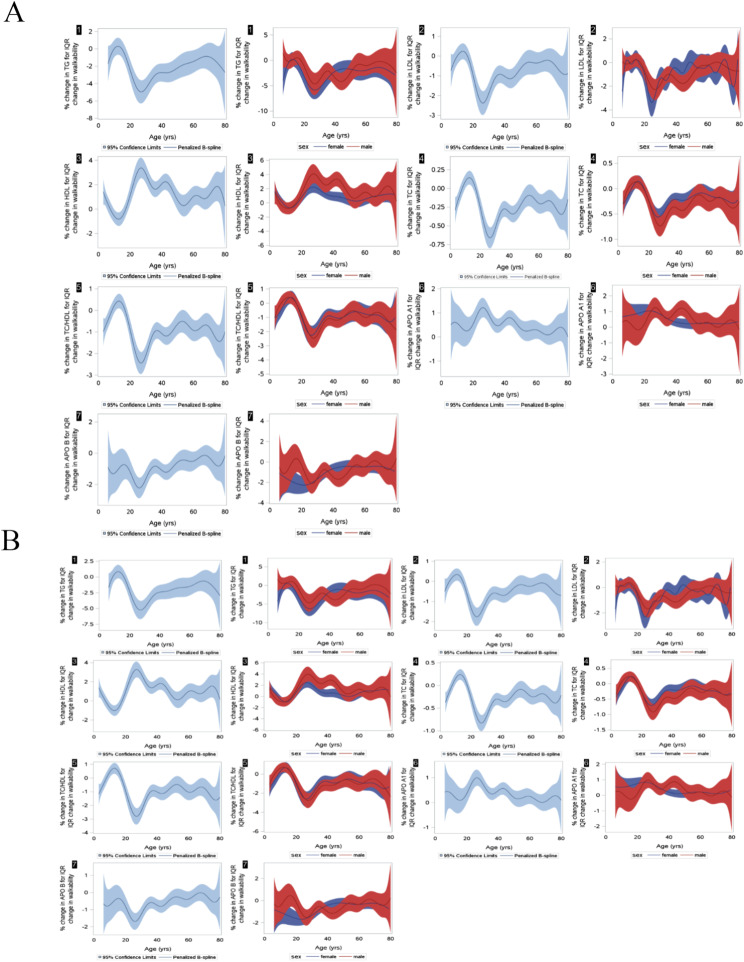
**a** Percent change in lipid biomarkers* for an IQR change in walkability (Can-ALE) by age and self-reported sex. Box numbers correspond to specific lipid: 1 represents TG (triglycerides), 2 represents LDL (low-density lipoprotein), 3 represents HDL (high-density lipoprotein), 4 represents TC (total cholesterol), 5 represents TC/HDL ratio, 6 represents APO AI (apolipoprotein AI), and 7 represents APO B (apolipoprotein B). **b** Percent change in lipid biomarkers* for an IQR change in walkability to neighborhood transit (ALE + T) by age and self-reported sex. *Box numbers correspond to specific lipid: 1 represents TG (triglycerides), 2 represents LDL (low-density lipoprotein), 3 represents HDL (high-density lipoprotein), 4 represents TC (total cholesterol), 5 represents TC/HDL ratio, 6 represents APO AI (apolipoprotein AI), and 7 represents APO B (apolipoprotein B)

ALE: In the overall population, significant inverse associations were found in unadjusted Model 1 for all lipids with walkability in unadjusted Model 1, except for HDL which had a significant positive (favorable) association and Apo A, which was not significant (Table 3, Fig. 1a). Results were similar with basic adjusted Model 2 where the inverse association was retained, with the exception, that TC became non-significant (Table 3). With full adjustment in Model 3, an IQR increase in ALE was associated with % (95%CI) changes in TG, HDL, and TC/HDL of -2.85 (-4.77, -0.93), 1.68 (0.93, 2.42), and − 1.68 (-2.8, -0.56), respectively (Table 3). Significant effects were largely restricted to adults (> 16 years old), whereas in the younger age group there were no significant associations between an active living environment and lipids in the fully adjusted model. Significant associations were more frequently seen in women than men. For women, fully adjusted significant associations were observed for TG: -3.69 (-5.57, -1.82), LDL: -1.71 (-3.04, -0.38), TC/HDL: -2.79 (-3.68, -1.90), as well as HDL: 2.74 (1.90, 3.58) and Apo A: 1.34 (0.78, 1.89) (Table 3).

**Table 3:** percent change (95%CI) in serum lipid biochemical markers per IQR increases in walkability (ALE) by age and sex groups and by three statistical models

Lipids^1^				Percent Change (95%CI)						*p*-value
		Age (years)		Model 1^2^		Model 2^3^		Model 3^4^		
			*n* ^5^	ALE	95%CI	ALE	95%CI	ALE	95%CI	
TG (mmol/L)		All	11,777	-3.86	(-5.97, -1.75)	-3.84	(-6.05, -1.63)	-2.85	(-4.77, -0.93)	**0.0036**
		*≤* 16	4434	-2.39	(-5.07, 0.29)	-0.88	(-3.67, 1.91)	-0.61	(-3.40, 2.18)	0.6699
		> 16	7343	-4.31	(-6.09, -2.52)	-4.11	(-6.03, -2.2)	-3.03	(-4.78, -1.28)	**0.0007**
		Males	5773	-4.15	(-7.12, -1.18)	-3.81	(-7.11, -0.5)	-2.46	(-5.40, 0.47)	0.0998
		Females	6004	-5.14	(-7.17, -3.10)	-4.27	(-6.38, -2.17)	-3.69	(-5.57, -1.82)	**0.0001**
LDL (mmol/L)		All	11,713	-1.28	(2.50, -0.05)	-1.34	(-2.57, -0.11)	-1.05	(-2.00.1, 00)	0.0507
		*≤* 16	4416	-0.18	(-1.82, 1.46)	-0.27	(-2.05, 1.52)	-0.40	(-2.22, 1.43)	0.6706
		> 16	7297	-1.19	(-2.27, -0.11)	-1.27	(-2.41, -0.14)	-1.03	(-2.08, 0.01)	0.0532
		Males	5743	-0.1	(-1.56, 1.37)	-0.43	(-1.95, 1.10)	-0.40	(-1.87, 1.08)	0.5959
		Females	5970	-2.28	(-3.71, -0.86)	-2.00	(-3.67, -0.32)	-1.71	(-3.04, -0.38)	**0.0120**
HDL (mmol/L)		All	26,063	1.48	(0.65, 2.31)	1.70	(0.98, 2.43)	1.68	(0.93, 2.42)	**< 0.001**
		*≤* 16	9675	1.00	(0.26, 1.75)	0.46	(-0.35, 1.27)	0.46	(-0.35, 1.27)	0.2641
		> 16	16,388	1.63	(0.80, 2.46)	1.88	(1.19, 2.56)	1.89	(1.18, 2.60)	**< 0.001**
		Males	12,807	0.80	(0.06, 1.53)	0.59	(-0.23, 1.41)	0.49	(-0.32, 1.31)	0.2339
		Females	13,256	2.50	(1.60, 3.40)	2.77	(1.96, 3.58)	2.74	(1.90, 3.58)	**< 0.001**
TC (mmol/L)		All	26,061	-0.56	(-1.10, -0.02)	-0.41	(-0.98, 0.17)	-0.37	(-0.86, 0.11)	0.1303
		*≤* 16	9674	0.10	(-0.44, 0.65)	-0.02	(-0.63, 0.58)	-0.09	(-0.70, 0.53)	0.7828
		> 16	16,387	-0.60	(-1.11, -0.08)	-0.47	(-0.99, 0.05)	-0.28	(-0.75, 0.19)	0.2480
		Males	12,806	-0.29	(-0.94, 0.36)	-0.31	(-0.99, 0.38)	-0.21	(-0.87, 0.44)	0.5209
		Females	13,255	-0.51	(-1.32, 0.3)	-0.16	(-1.01, 0.70)	-0.15	(-0.85, 0.54)	0.6671
TC/HDL		All	26,061	-0.57	(-1.76, 0.63)	-0.72	(-1.87, 0.44)	-1.06	(-2.32, 0.20)	0.1001
		< 16	9674	-0.49	(-1.94, 0.96)	-0.71	(-2.09, 0.67)	-0.86	(-2.08, 0.36)	0.1001
		> 16	16,387	-3.17	(-6.83, 0.48)	-3.05	(-7.19, 1.10)	-5.22	(-10.17, -0.28)	**0.0393**
		Males	12,806	-0.38	(-2.13, 1.37)	-0.76	(-2.42, 0.89)	-0.96	(-2.71, 0.78)	0.1686
		Females	13,255	-0.72	(-2.35, 0.91)	-0.65	(-2.26, 0.95)	-1.12	(-2.94, 0.70)	0.2802
Apo A (g/L)		All	6313	0.16	(-0.43, 0.75)	0.38	(-0.18, 0.95)	0.53	(-0.07, 1.14)	0.0840
		*≤* 16	2394	0.76	(-1.04, 2.56)	0.65	(-1.87, 3.18)	0.51	(-1.95, 2.97)	0.6869
		> 16	3919	0.16	(-0.4, 0.71)	0.36	(-0.15, 0.87)	0.50	(-0.04, 1.05)	0.0719
		Males	3106	-0.27	(-0.88, 0.33)	-0.61	(-1.28, 0.06)	-0.46	(-1.16, 0.25)	0.2022
		Females	3207	1.05	(0.51, 1.59)	1.21	(0.66, 1.77)	1.34	(0.78, 1.89)	**< 0.001**
Apo B (g/L)		All	6303	-1.22	(-2.31, -0.12)	-1.45	(-2.61, -0.3)	-0.93	(-1.93, 0.07)	0.0671
		*≤* 16	2393	-2.02	(-4.85, 0.82)	-1.31	(-3.78, 1.17)	-1.09	(-3.62, 1.43)	0.3977
		> 16	3910	-1.08	(-2.09, -0.08)	-1.25	(-2.31, -0.2)	-0.89	(-1.85, 0.08)	0.0735
		Males	3101	-0.59	(-1.91, 0.72)	-1.06	(2.58, 0.46)	-0.92	(-2.42, 0.59)	0.2339
		Females	3202	-1.9	(-3.25, -0.55)	-1.74	(-3.25, -0.22)	-1.15	(-2.38, 0.07)	0.0656

The bolded values indicate statistically significant changes at the alpha = 0.05 level (*p* < 0.05).

^1^- TG = triglycerides, LDL = low-density lipoprotein cholesterol, HDL = high-density lipoprotein cholesterol, TC = total cholesterol, TC/HDL = total cholesterol/high-density lipoprotein cholesterol, Apo A = apolipoprotein A, Apo B = apolipoprotein B

^2^- Model 1 is unadjusted

^3^-Model 2 is adjusted based on sex, age, race/culture, income, education, cycle and place of residence

^4^-Model 3 is Model 2 + adjustment for current smoking, environmental tobacco smoke, alcohol, and PM_2.5_

^5^ All 3 models have the same sample size. Missing values were assigned to a category and included in analysis

**Table 4:** Percent change (95%CI) in serum lipid biochemical markers per IQR increases in walkability (ALE + T) by age and sex groups and by three statistical models

Lipids^1^				Percent Change (95%CI)						*p*-value
		Age (years)		Model 1^2^		Model 2^3^		Model 3^4^		
			*n*	ALE + T	95%CI	ALE + T	95%CI	ALE + T	95%CI	
TG (mmol/L)		All	11,777	-3.97	(-6.23, -1.72)	-4.1	(-6.43, -1.76)	-3.12	(-5.24, -1.01)	**0.0037**
		*≤* 16	4434	-2.4	(-4.98, 0.19)	-0.98	(-3.68, 1.71)	-0.73	(-3.41, 1.94)	0.5918
		> 16	7343	-4.39	(-6.27, -2.51)	-4.33	(-6.34, -2.32)	-3.26	(-5.16, -1.36)	**0.0008**
		Males	5773	-4.58	(-7.78, -1.38)	-4.41	(-7.99, -0.83)	-3.1	(-6.43, 0.23)	0.0683
		Females	6004	-4.74	(-6.9, -2.57)	-3.99	(-6.22, -1.76)	-3.5	(-5.44, -1.55)	**0.0004**
LDL (mmol/L)		All	11,713	-1.19	(-2.49, 0.1)	-1.3	(-2.58, -0.01)	-1	(-2.09, 0.09)	0.0727
		*≤* 16	4416	-0.24	(-1.86, 1.39)	-0.43	(-2.2, 1.33)	-0.48	(-2.28, 1.32)	0.5995
		> 16	7297	-1.11	(-2.23, 0.02)	-1.21	(-2.39, -0.02)	-0.95	(-2.04, 0.13)	0.0856
		Males	5743	0.02	(-1.54, 1.58)	-0.32	(-1.92, 1.27)	-0.31	(-1.85, 1.22)	0.6890
		Females	5970	-2.23	(-3.76, -0.7)	-1.98	(-3.76, -0.21)	-1.69	(-3.10, -0.28)	**0.0188**
HDL (mmol/L)		All	26,063	1.44	(0.58, 2.31)	1.71	(0.96, 2.46)	1.68	(0.91, 2.45)	**< 0.001**
		*≤* 16	9675	0.75	(0, 1.5)	0.22	(-0.6, 1.04)	0.23	(-0.59, 1.05)	0.5822
		> 16	16,388	1.6	(0.73, 2.46)	1.9	(1.19, 2.62)	1.93	(1.20, 2.66)	**< 0.001**
		Males	12,807	0.79	(0.03, 1.54)	0.57	(-0.27, 1.4)	0.47	(-0.36, 1.31)	0.2663
		Females	13,256	2.47	(1.5, 3.43)	2.8	(1.94, 3.67)	2.79	(1.89, 3.68)	**< 0.001**
TC (mmol/L)		All	26,061	-0.62	(-1.19, -0.04)	-0.45	(-1.05, 0.15)	-0.41	(-0.92, 0.10)	0.1138
		*≤* 16	9674	0.11	(-0.43, 0.66)	0	(-0.6, 0.61)	-0.04	(-0.65, 0.57)	0.8903
		> 16	16,387	-0.66	(-1.2, -0.11)	-0.5	(-1.04, 0.05)	-0.29	(-0.79, 0.21)	0.2546
		Males	12,806	-0.34	(-1.04, 0.35)	-0.34	(-1.07, 0.4)	-0.23	(-0.94, 0.48)	0.5251
		Females	13,255	-0.54	(-1.38, 0.31)	-0.16	(-1.04, 0.72)	-0.14	(-0.86, 0.57)	0.6911
TC/HDL		All	26,061	-0.48	(-1.87, 0.9)	-0.68	(-2.02, 0.65)	-1.11	(-2.56, 0.35)	0.1355
		< 16	9674	-0.96	(-4.65, 2.74)	-0.61	(-1.99, 0.77)	-1	(-2.49, 0.50)	0.1922
		> 16	16,387	-2.56	(-6.92, 1.81)	-2.59	(-7.59, 2.42)	-4.71	-10.38, 0.96)	0.1042
		Males	12,806	-0.36	(-2.37, 1.65)	-0.82	(-2.74, 1.09)	-1.15	(-3.18, 0.88)	0.2678
		Females	13,255	-0.59	(-2.49, 1.31)	-0.51	(-2.39, 1.36)	-1	(-3.09, 1.08)	0.3468
Apo A (g/L)		All	6313	0.15	(-0.48, 0.78)	0.4	(-0.2, 1.01)	0.56	(-0.08, 1.20)	0.0856
		*≤* 16	2394	0.64	(-1.37, 2.65)	0.38	(-2.66, 3.42)	0.24	(-2.72, 3.19)	0.8747
		> 16	3919	0.14	(-0.44, 0.72)	0.39	(-0.16, 0.93)	0.54	(-0.03, 1.12)	0.0646
		Males	3106	-0.27	(-0.92, 0.39)	-0.62	(-1.34, 0.11)	-0.46	(-1.22, 0.29)	0.2311
		Females	3207	1.06	(0.48, 1.64)	1.26	(0.68, 1.84)	1.41	(0.82, 1.99)	**< 0.001**
Apo B (g/L)		All	6303	-1.17	(-2.36, 0.01)	-1.5	(-2.74, -0.27)	-0.95	(-2.03, 0.13)	0.0848
		*≤* 16	2393	-2.17	(-5.29, 0.96)	-1.2	(-4.03, 1.62)	-0.98	(-3.80, 1.85)	0.4985
		> 16	3910	-1.02	(-2.09, 0.04)	-1.25	(-2.37, -0.12)	-0.85	(-1.89, 0.19)	0.1080
		Males	3101	-0.52	(-1.96, 0.93)	-1.06	(-2.7, 0.57)	-0.92	(-2.55, 0.71)	0.2695
		Females	3202	-1.88	(-3.32, -0.44)	-1.78	(-3.39, -0.17)	-1.17	(-2.46, 0.13)	0.0775

The bolded values indicate statistically significant changes at the alpha = 0.05 level (*p* < 0.05).

^1^- TG = triglycerides, LDL = low-density lipoprotein cholesterol, HDL = high-density lipoprotein cholesterol, TC = total cholesterol, TC/HDL = total cholesterol/high-density lipoprotein cholesterol, Apo A = apolipoprotein A, Apo B = apolipoprotein B

^2^- Model 1 is unadjusted

^3^-Model 2 is adjusted based on sex, age, race/culture, income, education, cycle and place of residence

^4^-Model 3 is Model 2 + adjustment for current smoking, environmental tobacco smoke, alcohol, and PM_2.5_

ALE + T: In the overall population, significant inverse associations with walkability including transit were found in the unadjusted model for TG, TC, and TC/HDL (Table 3b, Fig. 1b). There was also a positive and significant association with HDL. Inverse associations were observed in the fully adjusted model with TG and TC/HDL, and a positive association was seen with HDL. Compared to children, favourable associations were more frequent in the adults with unadjusted associations seen between walkability and TG, HDL, TC, and TC/HDL. Adjusted associations were with TG, HDL, and TC/HDL with percent increases for an IQR in ALE + T of -3.30 (-5.16, -1.36), 1.93( 1.20, 2.66), and − 1.80 (-2.82, -0.73), respectively (Table 4). Females living in more walkable neighborhoods had lower TG, LDL, TC/HDL, and higher Apo A and HDL (Table 4).

In addition to stratification by age and sex to illustrate effect modification, we we included age and sex interactions with walkability in a comprehensive regression model. Our analysis revealed significantly stronger effects of walkability on HDL, TC/HDL ratio, and Apo A in females compared to males (*p* = 0.0009, 0.0441, 0.0004 for ALE and the respective markers; *p* = 0.0015, 0.0178, 0.0006 for ALE + T and the biomarkers, respectively). We also found significantly stronger effects of walkability on HDL in age > 16 compared to age < 17 (*p* = 0.041 for ALE and *p* = 0.020 for ALE+).

Effect modification of the lipid-ALE association by age is shown in Fig. 2a and b. The percentage change in TG for an IQR increase in walkability approximated zero in children between 10 and 15 years old. The largest percentage decrease in TG, 4–5%, was observed between the ages of 20 and 30 years old. Between the ages of 25 and 65 years old, effect sizes progressively decreased. A similar pattern was observed with LDL, TC, and TC/HDL. Apo β also had a similar pattern with the nadir at about 25 years and thereafter effect sizes decreasing up to about 65 years of age, but effect sizes were negative at all ages. For HDL, effect sizes were positive at all ages with a maximum at about 25 years old except for those between approximately 8 to 18 years old where the effect size was negative. For Apo A1, effect sizes ranged between 0 and 1 except for participants between 25 and 30 years old where the percentage increase was just above 1.

## Discussion

We found that neighbourhoods conducive to active living were associated with favourable markers of lipid metabolism among adult Canadians. Results were similar between analyses using ALE and ALE + T walkability indexes. Observed effects were more likely to be seen in adults compared to children, and in females compared to males. Consistent with our findings, Koohsari et al. [40] analyzed data from 7,171 adult participants of a cross-sectional health study in Alberta, Canada. Walkability, reflected by population density, intersections, and business destinations, was reported to decrease the number of metabolic syndrome risk factors. However, results of individual biomarkers were not reported, which prevented closer comparisons with the present study. A positive association with TG was found in the longitudinal component of the Multiethnic Study of Atherosclerosis (MESA) [15], but walkability was not significantly associated with TG in several other studies [13, 17, 18]. A cross-sectional analysis of data collected from 78,023 participants living in Toronto, Canada, found a positive correlation between the Walk Score^®^ and HDL with an adjusted β-coefficient of 0.052 (95% CI: 0.029, 0.075), *p* < 0.001, but there were no significant associations with TG or LDL in an adjusted analysis [12].

In contrast to our findings, Chandraboise et al. [13] followed 2,023 adults over 12 years in Australia and found a significant negative association between HDL and walkability, indicated by residential density, street connectivity, and daily living destinations, with a regression coefficient of -0.06 (95% CI: -0.10, -0.02). No effect was observed for TG. The authors invoked the possibility of increased access to unhealthy food, suggesting that differences in findings between studies could be related to differences in neighborhood characteristics, such as the proportion of accessible fast-food outlets. de Courreges et al. [17]. found no association between walkability, based on land use, street connectivity, and residential density, and TG, HDL, and LDL in a cross-sectional study of 3,267 adults in France. A longitudinal analysis of the 3,145 participants from the first two waves of the North West Adelaide Health Study (2000–2003 and 2005–2006) found no effect on a measure of dyslipidemia comprised of increased TG, decreased HDL, and being prescribed lipid-lowering agents [19]. A cross-sectional analysis of the Multiethnic Study of Atherosclerosis (MESA) found no association between the Street Smart Walk Score^®^ and levels of lipid biochemical markers, such as TG, TC, HDL, and LDL [15]. Hajna et al. [20]. analyzed CHMS cycle 1 only and found that increased walkability (using combined GIS-derived measures of neighborhood street connectivity, land use mix, and population density in a 500 m buffer of the residential six-digit postal code) was negatively associated with TC/HDL, but the interquartile linear trend was not significant (*p* = 0.08). The authors suggested that the lack of significant association may be related to the exclusion of those taking lipid-lowering medications. In the present study, there was an inverse association between ALE and Apo B in models 1 and 2 but not 3, especially for adults and females. Compared to this study, the much larger sample size of our study allowed more power to detect small changes in lipids. Previous studies did not consider Apo B [13, 15–20, 40]. To our knowledge, our study is the first to consider the influence of walkability on this major marker of CVD risk. Possible explanations for differences between studies apart from sample size include neighbourhood characteristics such as access to unhealthy food outlets, climate, and perhaps cultural or lifestyle or weather, which may modify the tendency to use active transport. Study design, whether cross-sectional or longitudinal, did not appear to be a determinant of whether findings were significant or not. Definitions of walkability varied but reflected population density and street connectivity.

### Age-related differences

The reasons for the observed age-related differential effect are unknown, and there is a paucity of previous information available to address this question because previous studies of walkability and blood lipids focused on adults [12, 17, 23, 25]. Perhaps through regular play and school activities, children are not as dependent as adults on housing and intersection density for their daily physical activities. Components of the active living index may affect children differently than adults. Compared to adults, access to shopping and business centers may be less important for children, but proximity to parks and playgrounds, as well as safe walking or cycling access to schools, may be more important [41, 42]. A Canadian study of 26,078 participants, aged 11–15 years old, found an inverse association between neighborhood walkability and the percentage of 11–15-year-olds who reported “≥60 min of daily moderate-to-vigorous physical activity [43]. Thus, the observed differences between children and adults regarding the association between ALE and lipid biomarkers may be due to differences in how neighborhood characteristics affect activity between these age groups.

### Gender/Sex-related differences

Associations between walkability and lipid metabolism biomarkers were more frequently seen in females compared to males. The reasons are unknown, but previous studies [43–47] have documented sex/gender-related differences in active living, which may in turn differentially influence the walkability-lipid association. In an American longitudinal study, females were twice as likely as males to live in the most walkable neighborhood category [47]. A systematic review of 36 studies from North America, Europe, Australia, and Asia published between 1995 and 2015 found that women were more likely than men to walk for leisure and for errands, but walking to catch transit or total walking were not consistently different [45]. Cohen et al. [46] discussed factors influencing the use of parks, including gender, age, income, disability, childcare responsibilities, and perceived safety, and carried out a large interview-based study in low-income neighborhoods in Los Angeles, USA. There were no significant gender-related differences in driving versus walking or cycling to a local park, but men used parks more often and for a longer duration than women. Also, women reported exercising less than men. A study carried out in suburban areas of metropolitan Vancouver, Canada, found that women were more likely than men to walk to school or work, and the odds ratio (OR) for recreation and park land was 0.76 (95% CI: 0.60–0.96) [44]. Jensen et al. [43] calculated the proportions of females and males using more and less walkable areas during weekdays. For females, the use of high/low walkable areas ratio was 2.1, whereas for males, it was 0.74. This indicates that increased walkability may have a larger behavioral effect on females than males. Assuming increased physical activity improves lipid profiles, increased walkability would tend to have a larger effect on females [43], which may explain the sex differences in our findings.

### Significance of findings

Our findings suggest that the built environment may influence lipid metabolism, which in turn has implications for cardiovascular health. We found that increased walkability was associated with higher HDL among adults. An inverse association between HDL and the risk of atherosclerotic CVD has been documented in epidemiologic studies, but interventions to increase HDL have not demonstrated a reduction in risk, indicating that the association may not be causal [48, 49]. We also found that TG were lower among adults living in more walkable neighborhoods. Previous meta-analyses have demonstrated significant positive associations between elevated TG and CVD, but a causal relation has not been universally accepted [50, 51]. Elevated TC/HDL is another predictor of CVD that we found to be favorably associated with increased walkability [52]. Apo B and LDL cholesterol are considered major risk factors for coronary artery disease, with Apo B having a greater predictive value [8, 22, 23]. We found negative associations with LDL and Apo B, although only the former was statistically significant. The more favourable levels of markers of lipid metabolism related to an increase in the active living environment index suggest that neighborhoods more conducive to physical activities, such as walking, may reduce the risk of cardiovascular disease.

Concerning cardiovascular disease risk factors apart from dyslipidemia, we and others have reported that increased walkability may favourably influence glucose metabolism [12, 13, 34]. Together, these observations support the hypothesis that increased walkability may reduce biomarkers of cardiovascular disease risk.

### Mediators of the walkability-lipid association

Presumably the links between walkability and improved CVD biomarkers include increased physical activity and weight loss. Neighbourhoods with a walkable built environment have been positively correlated with increased walking [53] and physical activity [13]. The ALE-Index was found to be modestly correlated with reports of walking to work (*R* = 0.47) and strongly correlated with actively getting to work by either walking, cycling or taking public transport (*R* = 0.78) [54]. The resulting increase in physical activity and its intensity could be conductive to weight loss, and a decreased risk of becoming overweight/obese as shown in an American study [47, 55].

### Strengths and limitations

Strengths of the present study include large multi-year samples of Canadian participants representative of the national population with objective measures of “active living” neighbourhoods and the availability of individual participant characteristics. We found novel associations between walkability and lipid metabolism in adults, and more so in women. We also included children of at least three years old into our analyses, as many previous analyses focused on adults only [12, 17, 18, 56] or pre-teens [24, 25]. Our findings were relatively consistent across different statistical models. We adjusted for PM_2.5_ since it has been associated with lipid levels [57] and would be influenced by the built environment. Additionally, previous studies often focus on a few lipid profile biomarkers [13, 15–18, 20, 40], whereas our study included seven lipid biochemical markers including Apo B, one of the major lipid risk factors for CVD.

A limitation of the study is its cross-sectional design which does not allow for determination of the causal directionality of the association. It is possible that people who are healthier, with more favourable lipid biomarkers, are more likely to move into more walkable neighborhoods. However, one previous longitudinal study found that walkability was associated with a lower future risk of being overweight [47] and another study found that higher walkability was associated with less weight gain [13], which clarifies the directionality of the association. Another limitation is that the present study did not have a measure of access to unhealthy food outlets (i.e., fast food restaurants and bakeries) which may reduce the observed benefits of facilitating walkability [19, 58]. We did not have a comprehensive dietary history but we expect that age, education, income, smoking and alcohol consumption should be related to diet, and we did adjust for these sociodemographic and lifestyle covariates. Finally, the statistical adjustments for age, sex, education and income may not fully control for other factors such as occupation, length of commute, and leisure time which could possibly influence the results.

## Concluding remarks and future perspectives

The current study found that those living in neighbourhoods conducive to active living have more favourable lipid profiles. This suggests that neighborhood walkability has the potential to improve the cardiovascular risk profile in adults, more so in females than males. Confirmatory longitudinal studies are recommended to confirm associations between neighbourhood walkability and the range of lipid markers used in this study, to further investigate the age and sex interactions we detected, and to investigate mechanisms explaining this association.

## Electronic supplementary material

Below is the link to the electronic supplementary material.

Supplementary Material 1

## Data Availability

No datasets were generated or analysed during the current study.

## Abbreviations

Apo A: Apolipoprotein A
Apo B: Apolipoprotein B
BMI: Body mass index
Can: ALE-Canadian Active Living Environments
CHMS: Canadian health measures survey
CI: Confidence intervals
CVD: Cardiovascular disease
DA: Dissemination area
HDL: High-density lipoprotein cholesterol
HR: Hazard ratio
IQR: Interquartile range
LDL: Low-density lipoprotein cholesterol
OR: Odds ratio
RR: Relative risk
SD: Standard deviation
TG: Triglycerides
TC: Total cholesterol
