# Supplementary material for: The association between neighbourhood walkability and blood lipids: a Canadian population study
Source: Lipids Health Dis. 2024 Sep 12;23:298. doi: 10.1186/s12944-024-02267-x (PMC11391640; doi:10.1186/s12944-024-02267-x)
Supplement: Supplementary file 1 — Supplementary Material 1 [file 12944_2024_2267_MOESM1_ESM.docx]

**Supplementary section:**

**Fig. S1a.** Percentage change in lipid biomarkers* (y-axis) for an IQR increase in walkability (ALE) (x-axis) estimated by three levels of statistical adjustment. Model 1 is unadjusted except for province of residence. Model 2 is adjusted for age, sex, race/culture, highest household education annual household income. cycle and province of residence. Model 3 is Model 2 plus adjustment for current smoking status, environmental tobacco smoke alcohol consumption and PM_2.5_. Plots are numbered 1 for the overall population, 2 for younger (≤16 years) participants, 3 for older (>16 years) participants, 4 for males and 5 for females.


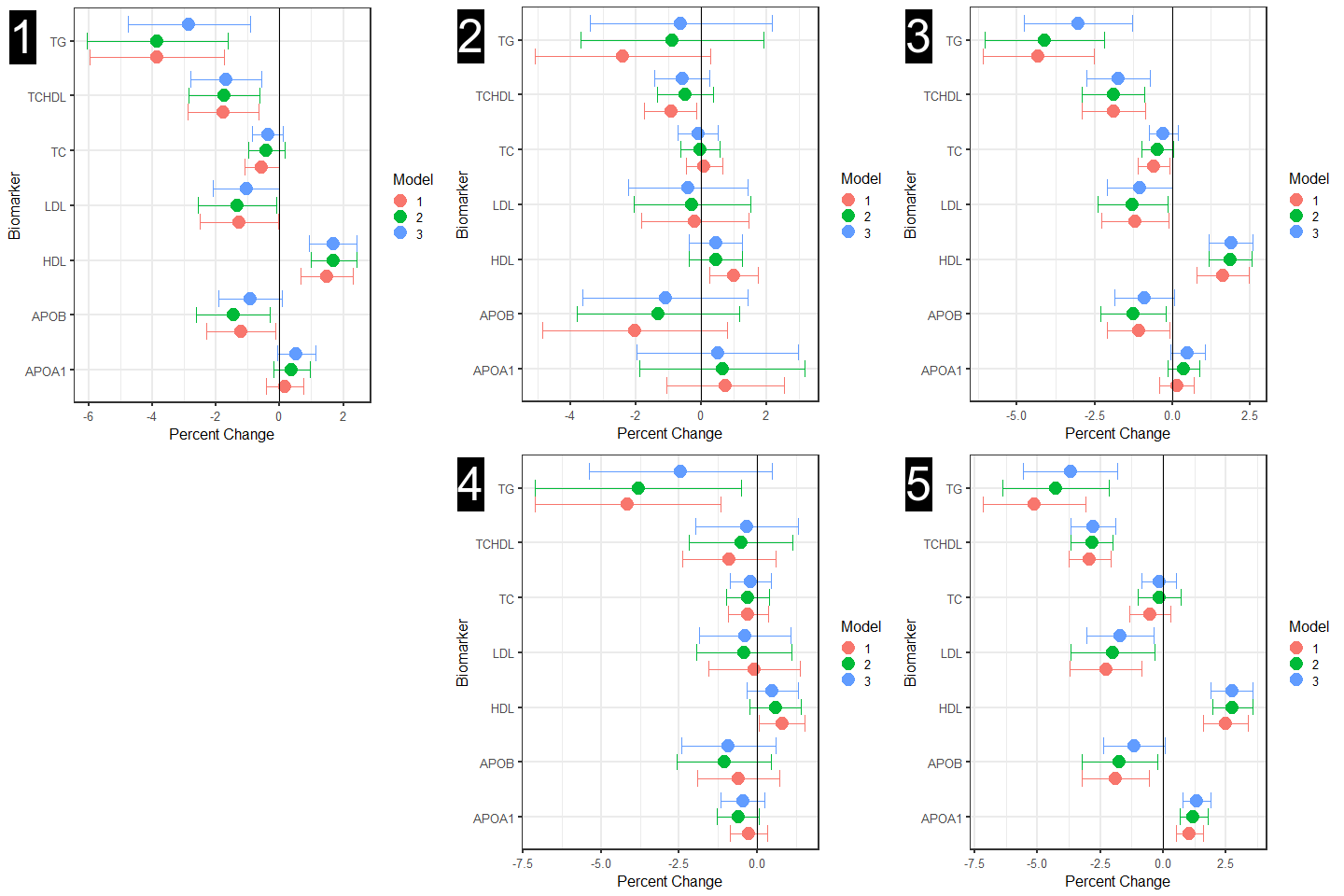


* Lipids are: TG (triglycerides), LDL (low-density lipoprotein cholesterol), HDL (high-density lipoprotein cholesterol), TC (total cholesterol), TC/HDL (total cholesterol/high-density lipoprotein cholesterol), APO A (apolipoprotein A), APO B (apolipoprotein B).

**Fig. S1b.** Percentage change in lipid biomarkers* (y-axis) for an IQR increase in walkability (ALE+T) (x-axis) estimated by three levels of statistical adjustment. Model 1 is unadjusted except for province of residence. Model 2 is adjusted for age, sex, race/culture, highest household education annual household income. cycle and province of residence. Model 3 is Model 2 plus adjustment for current smoking status, environmental tobacco smoke alcohol consumption and PM_2.5_. Plots are numbered 1 for the overall population, 2 for younger (≤ 16 years old) participants, 3 for older (> 16+ years old) participants, 4 for males and 5 for females.


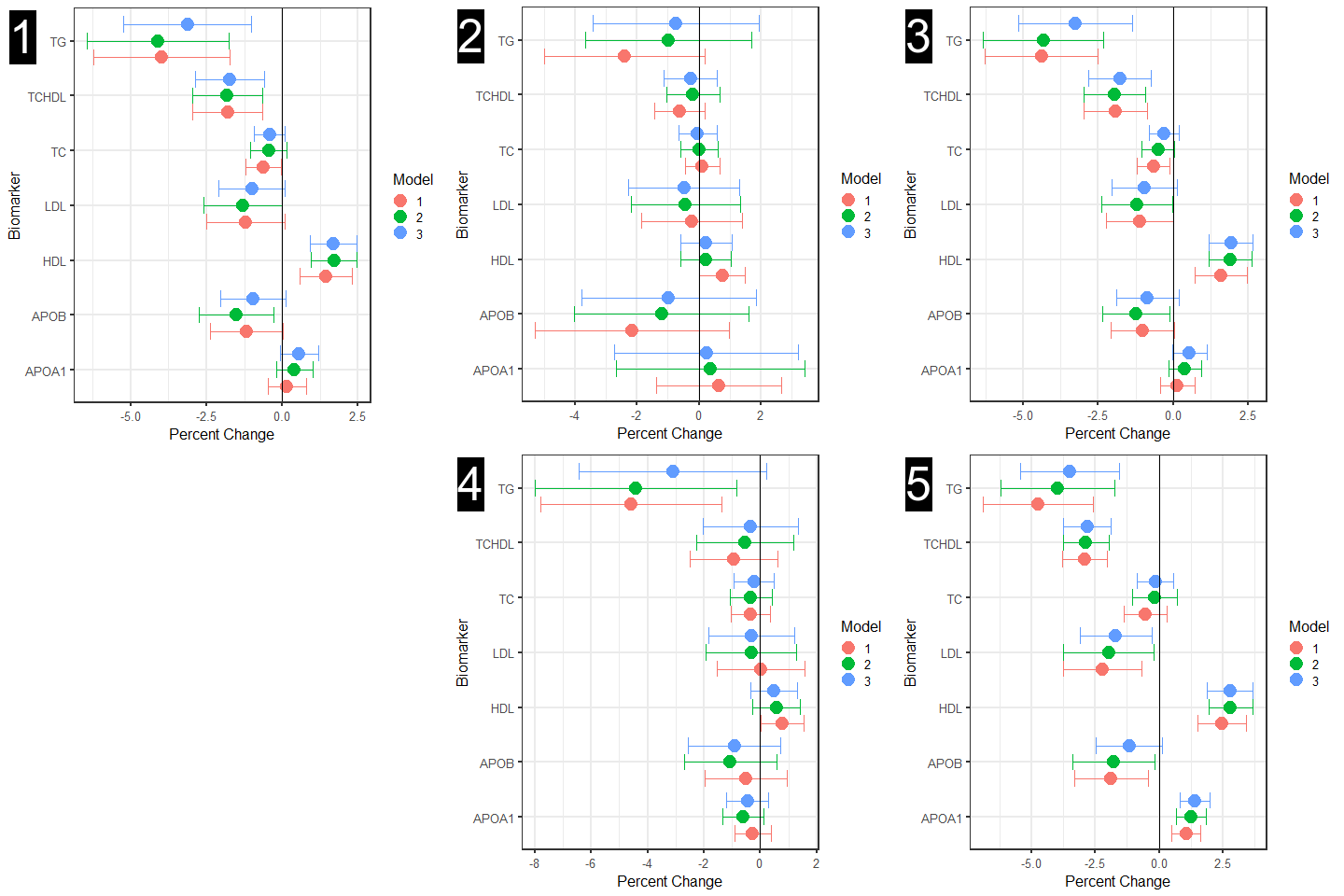


* Lipids are: TG (triglycerides), LDL (low-density lipoprotein cholesterol), HDL (high-density lipoprotein cholesterol), TC (total cholesterol), TC/HDL (total cholesterol/high-density lipoprotein cholesterol), Apo A (apolipoprotein A), Apo B (apolipoprotein B).

Table S1: Number (percent) of ALE and ALE+T by ALE_CLASS and ALE_TRANSIT_CLASS by score from 1(low) to 5 (high).

|  | **Class 1** | ALE | ALE + T |
| --- | --- | --- | --- |
|  | **Age group** |  |  |
|  | Age <16 years | 3335 (38.24) | 3303 (38.12) |
|  | Age >16 years | 5387 (61.76) | 5362 (61.88) |
|  | **Self-declared sex** |  |  |
|  | Male | 4299 (49.29) | 4266 (49.23) |
|  | Female | 4423 (50.71) | 4399 (50.77) |
|  | **Highest Household Educational attainment** |  |  |
|  | < Secondary school graduation | 495 (5.68) | 492 (5.68) |
|  | Secondary school education | 981 (11.25) | 977 (11.28) |
|  | Some post-secondary education | 233 (2.67) | 233 (2.69) |
|  | Post-secondary degree or diploma | 6742 (77.3) | 6699 (77.31) |
|  | Missing | 271 (3.11) | 264 (3.05) |
|  | **Total Annual Household Income** |  |  |
|  | First quartile (<$42,000) | 1958 (22.45) | 1954 (22.55) |
|  | Second quartile ($42,000-<$74,200) | 2185 (25.05) | 2163 (24.96) |
|  | Third quartile ($74,200-$115,000) | 2143 (24.57) | 2153 (24.85) |
|  | Fourth quartile (>$115,000) | 1946 (22.31) | 1913 (22.08) |
|  | Missing | 490 (5.62) | 482 (5.56) |
|  |  |  |  |
|  | **Class 2** |  |  |
|  | **Age group** |  |  |
|  | Age <16 years | 2689 (36.04) | 2798 (35.72) |
|  | Age >16 years | 4773 (63.96) | 5035 (64.28) |
|  | **Self-declared sex** |  |  |
|  | Male | 3654 (48.97) | 3811 (48.65) |
|  | Female | 3808 (51.03) | 4022 (51.35) |
|  | **Highest Household Educational attainment** |  |  |
|  | < Secondary school graduation | 428 (5.74) | 454 (5.8) |
|  | Secondary school education | 819 (10.98) | 869 (11.09) |
|  | Some post-secondary education | 221 (2.96) | 229 (2.92) |
|  | Post-secondary degree or diploma | 5750 (77.06) | 6021 (76.87) |
|  | Missing | 244 (3.27) | 260 (3.32) |
|  | **Total Annual Household Income:** |  |  |
|  | First quartile (<$42,000) | 1721 (23.06) | 1788 (22.83) |
|  | Second quartile ($42,000-<$74,200) | 1795 (24.06) | 1904 (24.31) |
|  | Third quartile ($74,200-$115,000) | 1924 (25.78) | 2006 (25.61) |
|  | Fourth quartile (>$115,000) | 1622 (21.74) | 1713 (21.87) |
|  | Missing | 400 (5.36) | 422 (5.39) |
|  |  |  |  |
|  | **Class 3** |  |  |
|  | **Age group** |  |  |
|  | Age <16 years | 2195 (37.02) | 2221 (37.57) |
|  | Age >16 years | 3734 (62.98) | 3690 (62.43) |
|  | **Self-declared sex** |  |  |
|  | Male | 2920 (49.25) | 2932 (49.6) |
|  | Female | 3009 (50.75) | 2979 (50.4) |
|  | **Highest Household Educational attainment** |  |  |
|  | < Secondary school graduation | 320 (5.4) | 320 (5.41) |
|  | Secondary school education | 663 (11.18) | 667 (11.28) |
|  | Some post-secondary education | 174 (2.93) | 171 (2.89) |
|  | Post-secondary degree or diploma | 4563 (76.96) | 4546 (76.91) |
|  | Missing | 209 (3.53) | 207 (3.5) |
|  | **Total Annual Household Income** |  |  |
|  | First quartile (<$42,000) | 1405 (23.7) | 1430 (24.19) |
|  | Second quartile ($42,000-<$74,200) | 1426 (24.05) | 1412 (23.89) |
|  | Third quartile ($74,200-$115,000) | 1453 (24.51) | 1428 (24.16) |
|  | Fourth quartile (>$115,000) | 1339 (22.58) | 1343 (22.72) |
|  | Missing | 306 (5.16) | 298 (5.04) |
|  |  |  |  |
|  | **Class 4** |  |  |
|  | **Age group** |  |  |
|  | Age <16 years | 869 (36.28) | 824 (36.27) |
|  | Age >16 years | 1526 (63.72) | 1448 (63.73) |
|  | **Self-declared sex** |  |  |
|  | Male | 1166 (48.68) | 1120 (49.3) |
|  | Female | 1229 (51.32) | 1152 (50.7) |
|  | **Highest Household Educational attainment** |  |  |
|  | < Secondary school graduation | 141 (5.89) | 133 (5.85) |
|  | Secondary school education | 286 (11.94) | 262 (11.53) |
|  | Some post-secondary education | 59 (2.46) | 58 (2.55) |
|  | Post-secondary degree or diploma | 1829 (76.37) | 1743 (76.72) |
|  | Missing | 80 (3.34) | 76 (3.35) |
|  | **Total Annual Household Income:** |  |  |
|  | First quartile (<$42,000) | 576 (24.05) | 545 (23.99) |
|  | Second quartile ($42,000-<$74,200) | 578 (24.13) | 555 (24.43) |
|  | Third quartile ($74,200-$115,000) | 591 (24.68) | 562 (24.74) |
|  | Fourth quartile (>$115,000) | 520 (21.71) | 488 (21.48) |
|  | Missing | 130 (5.43) | 122 (5.37) |
|  |  |  |  |
|  | **Class 5** |  |  |
|  | **Age group** |  |  |
|  | Age <16 years | 587 (37.75) | 529 (38.28) |
|  | Age >16 years | 968 (62.25) | 853 (61.72) |
|  | **Self-declared sex:** |  |  |
|  | Male | 768 (49.39) | 678 (49.06) |
|  | Female | 787 (50.61) | 704 (50.94) |
|  | **Highest Household Educational attainment:** |  |  |
|  | < Secondary school graduation | 94 (6.05) | 79 (5.72) |
|  | Secondary school education | 200 (12.86) | 174 (12.59) |
|  | Some post-secondary education | 38 (2.44) | 34 (2.46) |
|  | Post-secondary degree or diploma | 1191 (76.59) | 1066 (77.13) |
|  | Missing | 32 (2.06) | 29 (2.1) |
|  | **Total Annual Household Income:** |  |  |
|  | First quartile (<$42,000) | 385 (24.76) | 328 (23.73) |
|  | Second quartile ($42,000-<$74,200) | 378 (24.31) | 328 (23.73) |
|  | Third quartile ($74,200-$115,000) | 383 (24.63) | 345 (24.96) |
|  | Fourth quartile (>$115,000) | 354 (22.77) | 324 (23.44) |
|  | Missing | 55 (3.54) | 57 (4.12) |
|  |  |  |  |
|  |  |  |  |

Table S2: Number (%) of participants by Population size and ALE_CLASS *.

|  |  |  | **Population Size** |  |  |
| --- | --- | --- | --- | --- | --- |
| **ALE CLASS** | **Small (< 50,000)** | **Medium (50,000-250,000)** | **Large (250,000-1,000,000)** | **Very Large (1,000,000-5,000,000)** | **Megacity (> 5,000,000)** |
| **ALE 1** | 2877 (32.95) | 1541 (32.39) | 1166 (32.31) | 2057 (33.69) | 1024 (35.8) |
| **ALE 2** | 2661 (30.48) | 1426 (29.98) | 1075 (29.79) | 1826 (29.91) | 845 (29.55) |
| **ALE 3** | 1942 (22.24) | 1099 (23.1) | 848 (23.5) | 1413 (23.14) | 609 (21.29) |
| **ALE 4** | 778 (8.91) | 435 (9.14) | 337 (9.34) | 504 (8.25) | 218 (7.62) |
| **ALE 5** | 473 (5.42) | 256 (5.38) | 183 (5.07) | 306 (5.01) | 164 (5.73) |

- Metropolitan Census Area population or census agglomeration population <https://search.app/JYiTiT22p7QGFC5Y8>
